# Dynamics and Considerations in the Determination of the Excretion of Gluten Immunogenic Peptides in Urine: Individual Variability at Low Gluten Intake

**DOI:** 10.3390/nu13082624

**Published:** 2021-07-29

**Authors:** Laura Coto, Carolina Sousa, Angel Cebolla

**Affiliations:** 1 Biomedal S.L., 41900 Seville, Spain; laura.coto@biomedal.com; 2 Human Nutrition and Food Science Doctoral Program, University of Granada, 18011 Granada, Spain; 3 Department of Microbiology and Parasitology, Faculty of Pharmacy, University of Seville, 41012 Seville, Spain; csoumar@us.es

**Keywords:** gluten immunogenic peptides, gluten excretion urine, gluten-free diet monitoring, celiac disease

## Abstract

Background: A lifelong strict gluten-free diet is the only available treatment for celiac disease, but total exclusion of gluten is difficult to achieve. The aim of this study was to determine the range of time and the amount of gluten immunogenic peptides (GIP) excreted in urine after specific gluten ingestions. Methods: 20 healthy participants followed the same diet for 12 days in which 50 mg and 2 g of gluten were ingested and all the urinations were collected. GIP were analyzed by lateral flow immunoassay (LFIA) tests and quantified using an LFIA reader. Results: GIP were detected in 15% and 95% of participants after 50 mg and 2 g gluten intakes, respectively. The higher frequency and concentration of GIP was found between 6 and 9 h after both gluten ingestions. The ranges of detection were 3–12 h (50 mg) and 0–15 h (2 g). Conclusions: An increase in the frequency of urine tests may be a suitable approach to avoid false negative results. The use of the LFIA test in three urine samples collected at different times may show a sensitivity of 19.6% for a gluten ingestion like 50 mg, increasing to 93% after 2 g consumption.

## 1. Introduction

Celiac disease (CD) is a chronic systemic immune-mediated disease triggered by the ingestion of dietary gluten in genetically predisposed individuals with the human leukocyte antigen, HLA-DQ2, and/or HLA-DQ8 haplotypes [1]. The clinical presentation of CD is extremely variable, ranging from typical gastrointestinal symptomatology to extraintestinal symptoms or have no symptoms at all. Importantly, extraintestinal symptoms comprise a substantial proportion of the clinical manifestations of CD such as dermatitis herpetiformis, arthritis, neurological symptoms, anaemia, osteopenia, osteoporosis, tooth enamel defects, aphthous stomatitis, hypertransaminasemia, etc. [1,2,3]. The pathogenesis of CD involves structural changes in the small intestinal mucosa and intraepithelial lymphocyte infiltration when gluten immunogenic peptides (GIP) resistant to digestive enzymes cross the epithelial barrier to the lamina propria, leading to the activation of both innate and adaptive immune responses [2,4].

Currently, the only treatment available for CD is a lifelong gluten-free diet (GFD). Strict adherence to the GFD is crucial to reverse the clinical manifestations and to prevent long-term complications [1,3,5,6,7]. However, a diet with the total exclusion of gluten is challenging for most patients, who need high levels of discipline and motivation [1]. Moreover, GFD is more expensive, less palatable, and imposes social constraints, such as when dining out and traveling [8,9,10]. Consequently, a substantial number of patients with CD, especially those who are asymptomatic, commit diet transgressions and they are at risk of developing histological lesions and complications as a result of their condition [11]. The reported rates of GFD adherence range between 12% and 90% in adults [11,12,13] and between 23% and 98% in children [14].

Although it has been described in the literature that a daily ingestion of less than 50 mg appears to be safe for most patients with CD [15], other authors have decreased this level to 30 mg to avoid intestinal mucosal abnormalities [16]. As there is a great diversity in gluten sensitivity among individuals [15], the establishment of a harmless threshold of daily gluten intake for the celiac population remains a troublesome task.

Gluten is an alcohol-soluble mixture of storage proteins, known as prolamins, of cereals such as wheat, rye, and barley [17]. These proteins are fundamental for dough formation in bakery products because of their viscoelasticity; however, their applications in the food industry are broader [18]. Wheat gluten prolamins, called gliadins and glutenins, are characterized by being rich in proline and glutamine amino acids, which make them resistant to hydrolysis by gastric and pancreatic enzymes [17]. As a result, an innumerable diversity of GIP is produced in the gastrointestinal tract, triggering an immune response in individuals with CD. In any case, most of the immunogenicity could be assigned to a limited number of gluten epitopes [19]; among the GIP containing the most active T cell epitopes of CD, the α-gliadin 33-mer peptide has been described as a paradigm of immunodominance [20].

There is limited evidence regarding gluten digestion, metabolism, and excretion mechanisms. As a dietary protein, gluten hydrolyzation occurs mainly in the small intestine by pancreatic enzymes, which break polypeptides into small peptides and amino acids that are transported through the intestinal barrier [21,22]. Furthermore, it has been described that a fraction of longer peptides resistant to the action of the peptidases can also cross the basolateral membrane of the enterocytes and reach portal circulation [21]. Several authors have reported the detection of GIP in the urine of patients with CD and healthy individuals using mass spectrometry and antibody-based methods [11,13,23,24,25,26,27,28]. Thus, they demonstrated that gluten-derived peptides enter the kidneys, and after the ultrafiltration process they are partially or totally excreted in the urine. It remains unknown if a proportion of these peptides is also reabsorbed and then metabolized or excreted using alternative pathways.

The use of GIP detection in urine has been developed as a direct test for GFD monitoring in contrast to the classical methods, rather than only detecting the consequences of diet transgressions [11,23]. Urine is an advantageous sample for disease monitoring, as it can be collected fully non-invasively, in large amounts, and repeatedly over long periods of time [29]. Urine is a complex matrix of different components, such as water, glucose, proteins, amino acids, and inorganic salts [29]. However, the usual low concentration of protein in urine and its heterogeneity within and between individuals complicate the determination of the specific moment of analyte excretion [29]. The aim of this study was to determine the individual variability and the dynamics and limit of detection (LoD) of urine GIP excretion after two different amounts of low/moderate gluten ingestion (50 mg and 2 g) by monitoring a significant number of participants with minimized diet variations.

## 2. Materials and Methods

### 2.1. Study Population

Between January 2020 and March 2020, 20 healthy volunteers were enrolled from circles of relatives of Biomedal S.L. (Seville, Spain) employees in collaboration with the research group of the University of Seville (Seville, Spain). The criteria for inclusion as healthy volunteers were: (1) participants who were >18 years old; (2) not been diagnosed with CD, non-celiac gluten sensitivity, and no food allergies, food intolerances, and other kinds of gastrointestinal diseases; (3) participants who were prepared to follow a strict diet; and (4) to have the determination and abilities for daily urine and stool collection. The exclusion criteria were as follows: (1) participants with associated pathologies or severe psychiatric diseases; and (2) participants who did not collect the samples properly on at least 70% of occasions.

All the subjects provided written informed consent to participate in the study, which was approved by the local ethics committee (n. 2381-N-19).

### 2.2. Study Design

**Figure 1:**
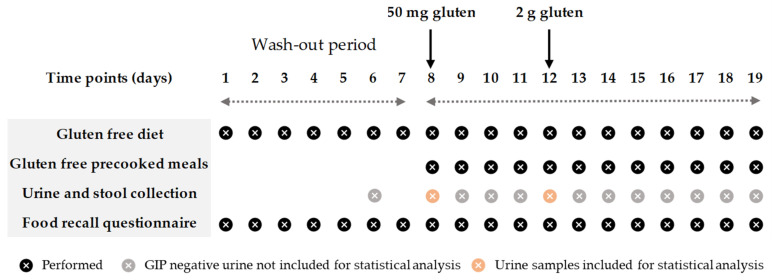
Study timeline.

The study involved all participants over a 19-day period. The first week was the wash-out stage, in which the participants had to follow a strict GFD. Two days before the first gluten ingestion, they were asked to collect one sample each of urine and feces to confirm the absence of dietary gluten (Figure 1). After the wash-out period, participants were provided with equivalent gluten-free lunch and dinner menus and gluten-free bread, which were supplied daily by the research team. The meals were consumed within the prescribed GFD. Two doses of gluten (50 mg and 2 g) were ingested in the morning (9:00) on days 8 and 12, respectively, and one sample of all the ordinary individual urinations and depositions (data published separately) were collected during the whole period (12 days in total). From the beginning to the end of the study, a food-recall questionnaire was used to assess GFD adherence and fluid intake, and the participants had to record the name and the quantity of the dishes that they consumed daily.

### 2.3. Gluten Administration

Gluten ingestions consisted of two doses of 50 mg and 2 g of powdered wheat gluten (El Granero Integral™; Biogran S.L., Madrid, Spain) encapsulated in “000” size gelatin caps (Your Supplements™, Bredbury, Stockport, England). The quantity selection was based on the minimum amount of gluten that, when eaten daily, could provoke histological changes in patients with CD [15] and an amount considered appropriate to observe the dynamic of excretion of GIP in urine. The gelatin caps were analyzed using GlutenTox^®^ ELISA Sandwich kit (Hygiena, Seville, Spain), based on G12 and A1 antibodies, to confirm the absence of gluten. Gluten estimation was calculated by analyzing several samples of maize starch Maizena™ (Unilever, London, England) spiked with the powdered gluten at different concentrations and analyzed using the GlutenTox^®^ ELISA Sandwich kit (Hygiena, Seville, Spain). Considering the results obtained (near 100% recovery), gluten doses for each subject were prepared using the total weight of the powdered gluten: 50 ± 5 mg and 2000 ± 5 mg in 1 and 4 caps, respectively.

**Figure 2:**
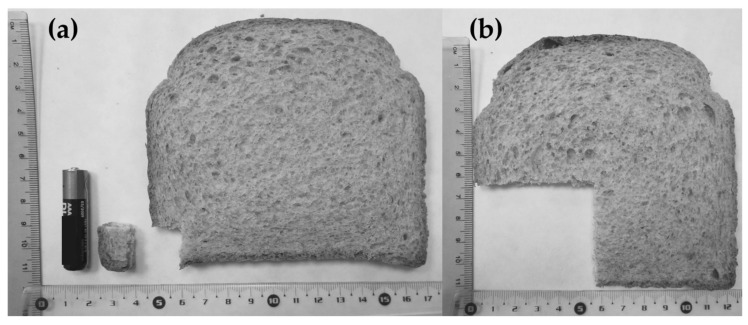
Small piece of bread from the slice representing 50 mg of gluten (**a**) and slice of bread corresponding to 2 g of gluten (**b**).

An equivalence calculation of the gluten dosages to bread portions was performed using the methodology described by Biagi et al. [30]. The slice of bread was 11 cm × 12 cm and weighted 30 g. Based on the nutritional composition given by the manufacturer, the whole slice contained 2.48 g of gluten. The corresponding amount of gluten in the bread slice was 0.6 g of slice for 50 mg of gluten (Figure 2a) and 24 g for 2 g of gluten (Figure 2b). A battery (AAA) was used as the standard for size comparison.

### 2.4. Meal Administration

All participants followed the same GFD during the gluten excretion period and were provided with ready-to-eat meals for lunch and dinner in addition to gluten-free certified bread (Beiker™, Dr. Schär, Postal BZ, Italy) to complete meals and for breakfast time. The diet was isocaloric and the ingestion of fresh fruits, unprocessed nuts, and gluten-free beverages was free of choice, depending on the energy requirements and habits of each participant. The meals were ordered from a catering company and were analyzed daily by the ISO17025 certified laboratory services of Biomedal S.L. (Seville, Spain), using GlutenTox^®^ ELISA Sandwich kit (Hygiena, Seville, Spain) to confirm the absence of gluten.

### 2.5. Urine Collection

Detailed instructions were given to all participants at the beginning of the study. The subjects were provided with all materials for urine collection, including specific plastic screw-capped containers, labels, cool bags, isothermal boxes, and cool packs. The participants were instructed to collect between 30 and 60 mL of each micturition and to write down the date and time of when they pass urine. All urine samples were preserved in isothermal boxes with cool packs at 4–8 °C and dropped off within 48 h of collection. All samples were stored at −20 °C until processing.

### 2.6. Urine Analysis

GIP qualitative results in urine were measured using a lateral flow immunoassay (LFIA) (iVYCHECK GIP Urine kit, Biomedal S.L., Seville, Spain) following the manufacturer’s recommendations. Defrosted urine samples were homogenized and mixed with a conditioning solution. Thereafter, 100 μL of the mixture was added to the immunochromatographic cassette and visual interpretation of the results was carried out after 30 min (recommended time for samples containing a low amount of GIP). A positive result was considered when the test line showed a red color, and the control line showed a green color. A negative result was considered when only the control line showed a green color. The LoD of the technique determined by visual inspection was 2 ng/mL.

The concentration of GIP in urine was also measured in the immunochromatographic strips after 30 min using the iVYCHECK Reader (Biomedal S.L., Seville, Spain). The validity of this method was previously described by Moreno et al. [23]. The reader was calibrated prior to urine analysis using the α-gliadin 33-mer peptide as a standard. The measuring range established for this method was: 1.56–25 ng GIP/mL urine. The results are expressed as ng GIP per mL of urine. Each sample was run in duplicate, and at least two different aliquots of each sample were tested.

### 2.7. Statistics

The results of the quantitative variables were expressed using the mean (SD) and median (IQR or range), and those of the categorical variables were expressed as absolute (N) and relative (%) frequencies. The goodness-of-fit to normality was calculated using the Shapiro–Wilk test. The Mann–Whitney U test was employed to compare quantitative variables in independent groups and for paired quantitative variables, the Wilcoxon test was used.

Only urines not later than 24 h post gluten ingestion were included for statistical analysis due to later urines from all participants giving a negative result. Ranges of time were established for the study of the dynamics of GIP excretion in intervals of 3 h. All samples from each participant collected in each range were clustered to obtain one result per participant. Any GIP+ sample indicated a total positive result.

Spearman’s correlation was used to calculate the association between the liquid consumption after gluten ingestion and the concentration of GIP in urine. Basic probability rules were used to obtain the diagnostic sensitivity of the studied techniques over a predetermined range of time with the different samples collected.

Statistical analyses were performed with IBM SPSS Statistics 25.0 for Windows (IBM Corp, Armonk, NY, USA). Statistical significance was set at *p* < 0.05.

## 3. Results

### 3.1. Subjects and Samples

**Figure 3:**
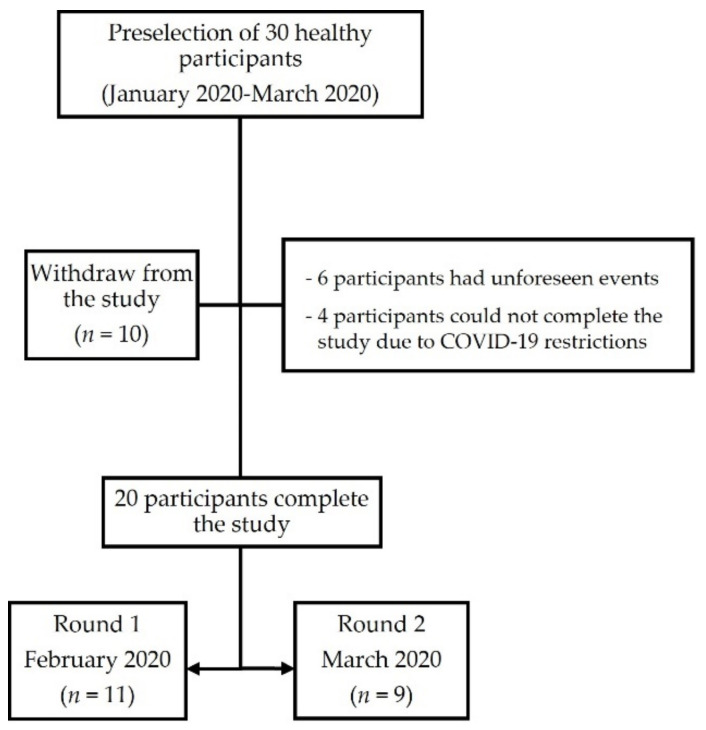
Flowchart of the study participants.

A total of 20 individuals, including 13 (65%) females and 7 (35%) males, completed the study after 10 dropouts from the preselection process due to unforeseen events (*n* = 6) and COVID-19 mobility restrictions (*n* = 4). The median age of participants was 30.5 years (IQR 24.7–34.0) (Figure 3).

None of the participants were declared to be diagnosed with a relevant disease or had been taking any probiotics or fiber supplements. One participant reported following a special fitness diet before the study. According to the food-recall completed, all participants were compliant with the prescribed GFD and the gluten dose ingestion. The average fluid intake per participant during the study period was 1.5 ± 0.6 L/day.

### 3.2. GIP Detection in Urine Samples

A total of 290 urine samples were collected from all participants during the 24 h after gluten ingestion, 142 corresponding to the 50 mg gluten dose, and 148 to the 2 g gluten dose. The remaining samples of the study were excluded for statistical analysis as they obtained GIP negative results. The medians of the number of samples collected per participant in the first 24 h were 7 (IQR 5–8) for the 50 mg intake and 7 (IQR 5.5–8.5) for the 2 g intake (Table 1).

GIP were detected in 4/142 (2.8%) of the urine samples up to 24 h after 50 mg gluten ingestion, corresponding to 3/20 (15%) participants. From these participants, GIP were detected in only one sample for two subjects and in two samples for one subject. Regarding the 2 g dose, 33.1% (49/148) of the urine samples were GIP+ during the 24 h of collection, corresponding to 19/20 (95%) of the participants. GIP+ samples were obtained in only one to two urinations for 10/19 participants (52.6%), in three to four urinations for 7/19 participants (36.8%), and in five to six urinations for 2/19 participants (10.5%).

GIP+ samples were found from the first to the fourth collected samples after 50 mg gluten ingestion. The detection of GIP in urine could be extended up to the eight urinations after the 2 g dose, with the third sample being where most participants (16/20; 80%) had GIP+ urine.

### 3.3. Time Course of GIP Excretion

**Figure 4:**
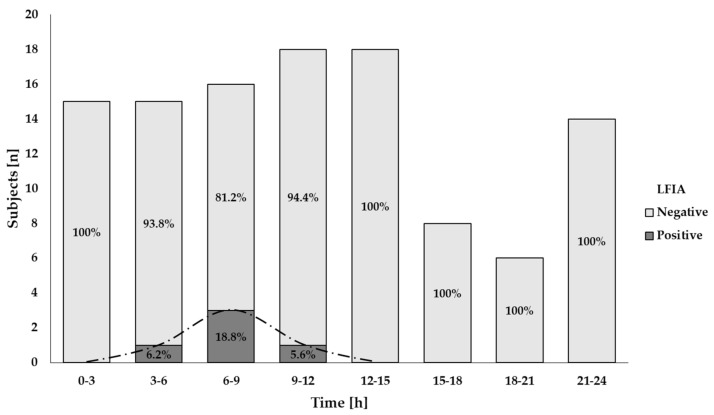
Results of qualitative analysis of GIP excretion in urine after 50 mg of gluten ingestion using a LFIA test. The trend of the GIP detection dynamics is represented by the dashed line.

**Figure 5:**
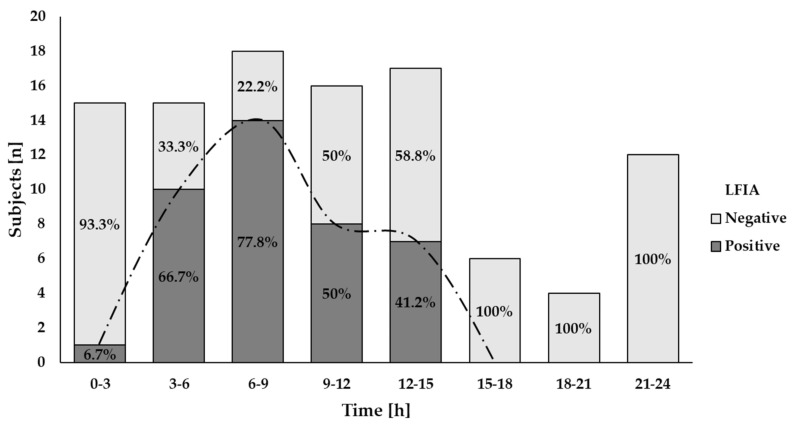
Results of qualitative analysis of GIP excretion in urine after 2 g of gluten ingestion using a LFIA test. The trend of the GIP detection dynamics is represented by the dashed line.

GIP were detected in urine samples collected in the first 3 h after 2 g gluten intake and between 3–6 h after 50 mg gluten ingestion (Figure 4 and Figure 5). The majority of GIP+ urine samples were found in the range of 6–9 h after both gluten doses (18.8% and 78.8%, respectively) (Figure 4 and Figure 5). As expected, the 2 g ingestion resulted in significant proportions of positive samples for a longer period (3–15 h) with rates between 41.2% and 78.8% (Figure 5). No positive results were found after 12 h and 15 h post ingestion for the 50 mg and the 2 g doses, respectively (Figure 4 and Figure 5).

Despite the variability observed among individuals, both gluten ingestions showed a comparable period for initial GIP detection (7.1 h (range 3.3–7.7)) for the 50 mg dose and 5.8 h (range 1.5–14.7) for the 2 g dose) (*p* = 0.285). A longer range of time for detectable GIP per participant was found in the larger gluten dose (0 (range 0–2.4) vs. 3.1 (range 0–8.8)) but without statistical significance (*p* = 0.180).

### 3.4. GIP Quantification in Urine

**Figure 6:**
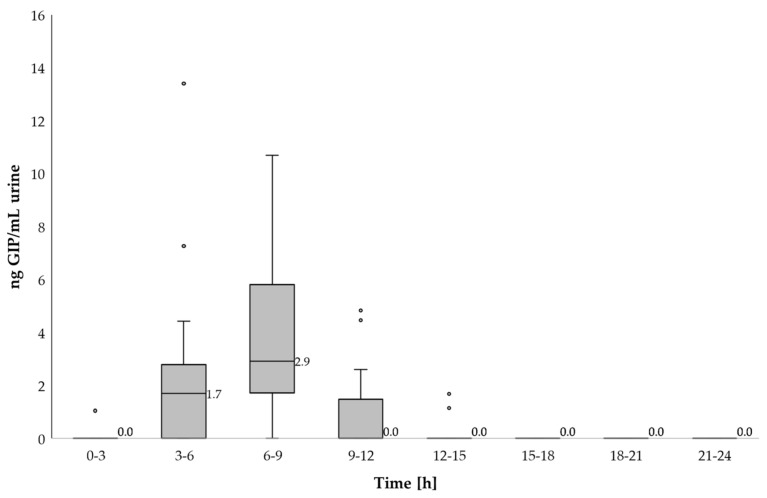
Dynamic of GIP excretion in urine after 2 g of gluten intake using a LFIA reader. Potential outliers are represented as dots.

In line with the time for GIP detection after gluten ingestion, higher concentrations of GIP were measured in the urine of most participants in the same period (6–9 h) using an LFIA reader. The median of GIP in this period was 0 ng GIP/mL urine (range 0–2.8) for the 50 mg dose and 2.57 ng GIP/mL urine (range 0–13.2) for the 2 g intake (Figure 6, Table 2).

**Figure 7:**
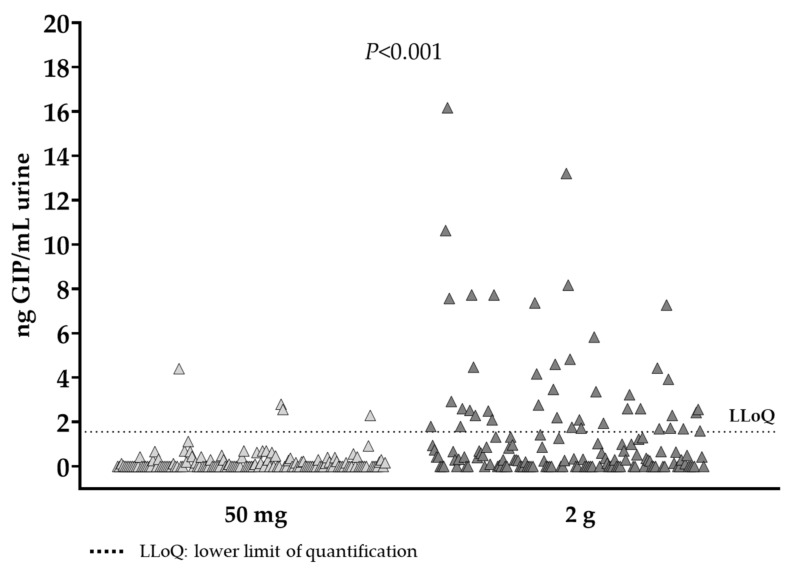
GIP detected in urine samples within 24 h after 50 mg and 2 g gluten ingestions.

However, the peak levels of GIP in urine were observed at different time periods; for the 50 mg dose, it was 4.4 ng GIP/mL, detected 3.3 h post ingestion and for the 2 g dose it was 16.17 ng GIP/mL, detected 5 h after gluten ingestion. Considering the period of GIP detection after both gluten doses (3–12 h) the median of GIP was 0 ng GIP/mL urine (range 0–4.4) for the 50 mg dose and 1.73 ng GIP/mL urine (range 0–16.17) for the 2 g intake with statistical differences between ingestions (*p* < 0.001) (Figure 7).

**Figure 8:**
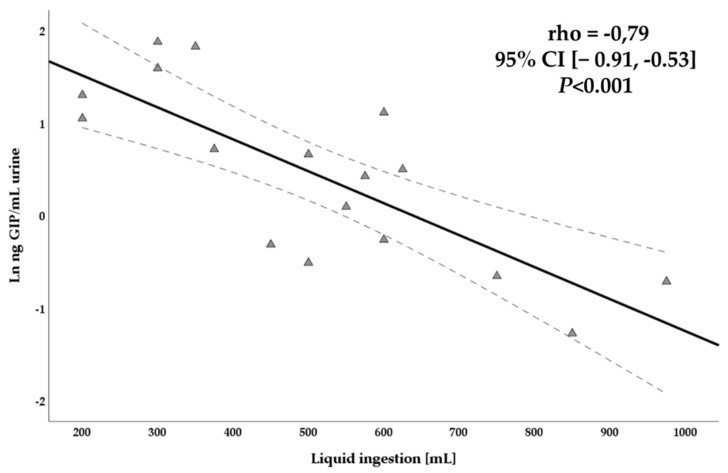
Scatterplot of liquid consumption after 12 h gluten intake and GIP concentration in urine using a LFIA reader.

We observed a significant negative correlation between the liquid consumption 12 h after gluten consumption and the levels of GIP in urine detected using an LFIA reader in the same period (rho = −0.79, 95% CI [−0.91, −0.53]; *p* < 0.001) (Figure 8). In contrast, no correlations were found between liquid consumption and urination frequency (rho = 0.119, 95% CI [−0.34, 0.53]; *p* = 0.62) and GIP concentrations and urination frequency (rho = −0.004, 95% CI [−0.45, 0.44]; *p* = 0.99).

### 3.5. Interindividual Variability in GIP Excretion

**Figure 9:**
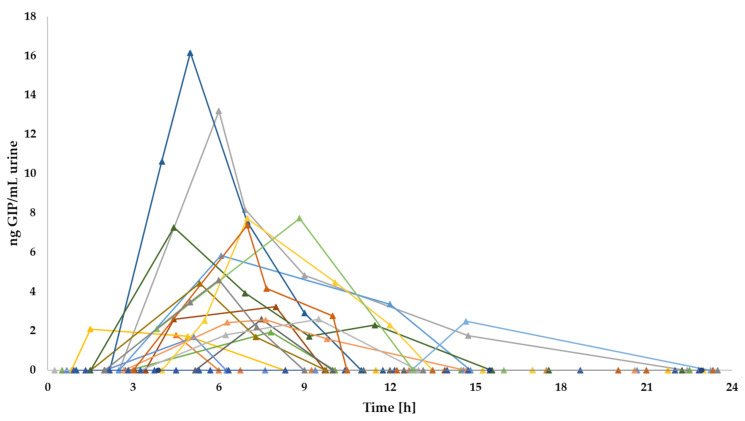
Individual GIP excretion patterns in urine after 2 g gluten ingestion.

Despite the limited period of GIP detection in urine after gluten intake, differences in GIP excretion were observed among individuals. Regarding the 50 mg gluten dose, one of three participants reached the peak of GIP concentrations in urine in the first 6 h post ingestion and two of three participants between 6 and 9 h post ingestion. Regarding the 2 g gluten dose, 6/20 (30%) participants reached the peak of GIP detection in the range of 0–6 h, 9/20 (45%) in the range of 6–9 h and 3/20 (15%) after 9 h post gluten ingestion (Figure 9). Although there were differences in GIP concentrations, GIP excretion patterns were similar in the participants with measurable excreted GIP after both gluten intakes. However, one participant (subject 6) obtained the peak of GIP concentration in the 2 g dose approximately 6 h later than the 50 mg dose. In general, no unusual patterns were observed, as most individuals showed a GIP elevation (3–9 h after gluten intake) followed by a decreasing tendency.

When the results were compared between sex, the higher GIP concentrations were seen in the group of females in both gluten intakes, however no statistical significance was observed between females and males in GIP detection (4.40 ng/mL vs. 2.50 ng/mL, respectively, for the 50 mg dose (*p* = 0.319); 2.69 ng/mL vs. 3.12 ng/mL, respectively, for the 2 g dose (*p* = 0.162)). Moreover, similar results were found between groups in terms of initial time of GIP detection (3.3 h vs. 7.38 h, respectively, for the 50 mg dose (*p* = 0.221); 6.22 h vs. 5.59 h, respectively, for the 2 g dose (*p* = 0.730)) and time range of GIP detection (0 h vs. 1.21 h, respectively, for the 50 mg dose (*p* = 0.480); 2.90 h vs. 3.59 h, respectively, for the 2 g dose (*p* = 0.688)).

### 3.6. Diagnostic Sensitivity of the LFIA Test

The LFIA test in urine samples demonstrated the capacity for GIP detection after a low gluten ingestion (50 mg). However, this amount of gluten was only detected in 3 out of 20 subjects (15%). In contrast, with a dose of 2 g of gluten, the sensitivity of the test increased to 95% of participants (19/20).

The theoretical probability of finding at least one GIP+ result for a single gluten ingestion was calculated considering the interval of time for GIP detection in both amounts of gluten, 3–12 h after ingestion (Table 3). The diagnostic sensitivity of the LFIA test to detect GIP in a unique urine sample from a small amount of gluten (50 mg) is 7%, which may increase to 13.5% when two samples are collected and to 19.6% in three samples collected. In the hypothetical situation of frequent gluten ingestion (i.e., 2 g), the sensitivity in a single sample may be 59%, reaching rates of 83.2% and 93.1% when two and three samples are collected, respectively.

## 4. Discussion

In the present study, we described the dynamics of excretion of GIP in urine samples of healthy subjects who ingested two small doses of gluten under controlled dietary conditions using an immunoassay method based on the anti-33-mer antibodies, G12 and A1 [23,31]. Our results confirmed that the LFIA test could detect a single ingestion of 50 mg of gluten in urine samples collected in a range of 3–10 h post ingestion, with most GIP excreted in a unique sample per participant. Equivalent results were obtained when the gluten ingestion was 40 times higher (2 g). GIP were detected for this gluten dose in the range of 1–15 h, with most of them obtained between 6 and 9 h post gluten ingestion.

Consistent with our data, Moreno et al. [23] were the first to demonstrate that the same LFIA method could detect the ingestion of 25 mg and 50 mg of gluten from processed bread in the urine of healthy volunteers. Their results revealed that GIP from those gluten doses were detectable 3–9 h post ingestion; however, the time of GIP disappearance after a normal gluten-containing diet was extended to 16–34 h compared to this study. They estimated that the time of excretion of gluten-derived peptides ranged from 1 to 2 days. In agreement with these data, other authors found an association between confirmed gluten exposure and GIP presence in urine within 36 h after ingestion in patients with CD [26,27]. Although the results exhibited a high interindividual variability, the interval between gluten consumption and GIP detection in urine was generally consistent, ranging from <4 to >24 h. Our results with more participants (*n* = 20) showed GIP detection in the first 15 h after the 2 g gluten challenge. Thus, depending on the amount of gluten consumed, the period of GIP detection may vary, with a positive trend between gluten consumption and GIP excretion. Nonetheless, it seems that the interval of time between 3 and 9 h post ingestion may be crucial for GIP detection, independent of the magnitude of gluten exposure. In any case, disagreement in the period to excrete all GIP could also vary depending on the type of ingested gluten, for instance, capsulated gluten in this study vs. other alternatives such as cookies, bread, or cereal bars that have been used in other studies [23,26,27].

Regarding the GIP concentration in urine, we found a significant variation in GIP content of samples collected 24 h after ingestion of 50 mg and 2 g gluten (*p* < 0.001). In this study, we observed that the higher the amount of gluten consumed, the more frequent GIP detection and quantification in urine. Nevertheless, interindividual variability was observed, with GIP medians of participants ranging from 2.3–4.4 ng/g for the 50 mg dose and from 1.7–9.1 ng/g for the 2 g dose. The study carried out by Moreno et al. [23] also showed slightly less differences in the maximum GIP content in urine collected after 25 and 50 mg of gluten ingestion (10–15 ng/mL vs. 15–20 ng/mL, respectively), however they pretreat the urine sample with solid phase extraction. Deviations between our results and those from previous studies could be due to the matrix containing the gluten used in the study and the methodology used for GIP quantification. 

The correlation between the amount of gluten consumed and the excretion of gluten-derived peptides has been previously described [11,23,26,27]. Generally, urine samples from healthy subjects under a normal gluten-containing diet showed a higher amount of GIP than in diet transgressions made by patients with CD. Indeed, most urine samples from these patients were detectable but were under the limit of quantitation [11,23,26,27]. Moreno et al. [23] reported GIP quantifications ranging from 6.5 to 600 ng/mL and 6.5 to 370 ng/mL in healthy adults and children, respectively, whereas GIP content in urines from patients with CD ranged from 9.27 to 78.12 ng GIP/mL and from 9.33 to 29.78 ng GIP/mL (in adults and children, respectively). Other authors reported significant differences in urine GIP concentrations between patients with CD under a GFD and de novo CD-diagnosed patients (average range 40.26 ng/mL vs. 80.31 ng/mL, *p* < 0.001) [11].

Although gluten consumption and diet composition were controlled in our study, urinary GIP excretion varied among participants. Urine composition can vary between individuals due to differences in biological factors, body size, physical exercise, environmental conditions, and fluid, salt, and high protein ingestion [32]. Moreover, the sample collection timing in relation to exposure, variation in the kinetics of elimination within and between individuals, and physicochemical properties of the urine matrix should be considered [33]. Hydration status plays a crucial role in variations in the urinary flow rate (volume of urine produced per unit time), and therefore in the concentrations of the biomarker in the study [33]. Although the mechanisms of GIP elimination in urine are currently unknown, our results showed a significant inverse correlation between liquid consumption and GIP concentration in urine (*p* < 0.001), as expected for a higher dilution of the urine peptides. However, we did not find a correlation between the urination frequency and GIP concentrations. Thus, it seems that urinary GIP detection may be affected by the amount of liquid ingested. On the other hand, some individuals may not absorb and excrete sufficient GIP in urine to be detected or a fraction of the absorbed GIP might go back to the portal circulation after their pass through the kidneys. Despite one of the participants of the present study obtaining negative results in all the urine samples collected after the 2 g gluten ingestion, GIP were significantly found in the stools coming from the same period, confirming the gluten exposure (data published separately). Another explanation could be that this participant missed the collection of one or more samples due to the tedious methodology employed in the study.

In a real-life scenario, following a strict GFD is a difficult task for patients with CD. Gluten is reported to be present in a significant percentage of foodstuffs [34]. Consequently, the frequency of diet transgressions is considerable, despite the assumed efforts of the patients [11,13,26,27,35]. Furthermore, it was suggested that inadvertent gluten ingestion may be more recurrent than intentional intake, not only when eating out, but also at home [12,26,27]. The main goal of this study was to comprehensively determine the pattern of GIP excretion in urine related to a single ingestion of a low amount of gluten, which is the expected situation for inadvertent-involuntary gluten exposure. This information will be valuable in providing more accurate guidance for the use of the GIP tests in patients with CD. On the basis of these results, future studies with the target population may build an effective protocol for urine sample collection to establish the algorithm of assessment of GFD adherence.

On the other hand, a recent publication with a cohort of 77 participants under a GFD for ≥2 years revealed that the urine LFIA test obtained a diagnostic sensitivity of 94.4% and negative predictive value of 96.9% in detecting mucosal damage when urine samples were collected on three different days, two of them over the weekend [11]. Other authors reported a rate of 69.8% of patients with at least one GIP+ in urine when they collected weekly samples on weekends over 4 weeks [13]. Thus, considering the short period of GIP detection in urine (3–12 h) after gluten intake and the variability in GIP excretion within and between individuals in our study, it seems that the increase in the frequency of tests may be a worthwhile approach to reduce the probability of false negative results due to punctual gluten consumptions [11,13]. In this scenario, the use of three LFIA tests in urine collected at different times during weekdays and weekends may reach a sensitivity of 19.6% for very low gluten intakes, such as 50 mg, while this sensitivity could increase to 93.1% with higher gluten exposures, such as 2 g.

Regarding the optimum time for urine collection, several circumstances need to be considered, such as the period with a higher rate of GIP detection: 3–12 h post ingestion, meals with a greater chance of gluten exposure (lunch and dinner), and the best time to obtain a concentrated urine sample in most individuals. Hence, it seems that the last urine in the night, or alternatively the first one in the morning, would meet most of these conditions. Routinely, first-morning urine samples are required for urinary analysis as a representative sample of the average urine of the day and because they have the highest concentration of peptides [33]. However, food ingestion occurs during the day, therefore, depending on the dynamics of excretion, analyte detection among subjects could fluctuate [33]. Alternations of first-morning urine and the last urine in the day may offer more probabilities to reveal a diet transgression made at lunch and dinner times. Moreno et al. [23] suggested the collection of 24-h total urine to increase the probability of GIP detection from a low amount of gluten ingestion; however, the complexity for patients is higher and urine samples with detectable amounts of GIP could be diluted, decreasing the concentration of the final sample.

The main limitations of this study were the inclusion of only healthy volunteers, and that maybe the sample size could be increased. The complexity of the study design, requiring a big effort from the volunteers, and the declaration of the COVID-19 pandemic make the recruitment process a difficult task. Furthermore, the inclusion of patients with CD had ethical concerns. Although it is generally believed that gluten metabolism is similar between patients with CD and healthy subjects, several aspects need to be addressed when the CD population is considered as they may present digestive alterations, intestinal permeability, and differences in the microbiota involved in the gluten degradation process [21,36,37]. In fact, it was described that patients with CD may have a higher proteolytic activity in the intestine leading to a gluten reduction in feces in comparison to healthy subjects and first-degree relatives on normal diet [37]. Moreover, a recent study confirmed that patients with CD consuming wheat excreted in urine a significantly higher diversity of gluten-derived peptides than healthy subjects, however differences the healing of the intestinal epithelia between patients with CD were not contemplated [28]. Thus, since the test for GIP detection in urine is intended for use by people suffering from CD and gluten-related disorders, future studies with these populations with similar gluten consumptions will confirm the compatibility of our results for the definition of clinical practice guidelines for the application of GIP in the monitoring of GFD.

The ability to capture a biomarker in a sample of urine is a noninvasive procedure that is convenient for almost all population [33]. Therefore, urinary GIP detection provides a supplemental tool to evaluate gluten exposure in individuals following a GFD. In conclusion, the results of this study will provide additional knowledge about gluten metabolism and GIP excretion, which could be useful to fine-tune the application of GIP determination in the follow-up of patients with CD and gluten-related disorders.

## Figures and Tables

**Table 1:** Individual characteristics of GIP excretion in urine within 24 h after 50 mg and 2 g gluten ingestions.

Urine GIP Excretion in 24 h														
	50 mg Gluten							2 g Gluten						
Participant	Samples	LFIA+	Time Median	Time Range	Peak Max GIP	GIP Median	GIP Range	Samples	LFIA+	Time Median	Time Range	Peak Max GIP	GIP Median	GIP Range
	*n*	*n*	h	h	h	ng/mL	ng/mL	*n*	*n*	h	h	h	ng/mL	ng/mL
1	7	0						6	1	4.50	0.00 (4.50)	4.50	1.80	0.00 (1.80)
2	7	0						8	6	6.00	5.00 (4.00–9.00)	5.00	9.10	13.23 (2.93–16.17)
3	4	0						6	3	7.88	3.25 (6.25–9.50)	9.50	2.20	0.80 (1.80–2.60)
4	8	0						9	4	8.54	6.50 (5.50–12.00)	7.00	3.50	5.43 (2.30–7.73)
5	6	0						4	1	14.67	0.00 (14.67)	14.67	2.50	0.00 (2.50)
6	4	1	3.33	0.00 (3.33)	3.33	4.40	0.00 (4.40)	4	3	6.33	5.00 (3.83–8.83)	8.83	4.92	5.63 (2.10–7.73)
7	15	0						18	1					
8	10	0						10	5	7.67	3.00 (7.00–10.00)	7.00	4.17	4.60 (2.77–7.37)
9	7	0						7	3	6.00	2.33 (5.00–7.33)	6.00	3.47	2.40 (2.20–4.60)
10	10	0						7	4	7.96	8.75 (6.00–14.75)	6.00	6.50	11.43 (1.77–13.20)
11	6	0						7	2	3.21	3.42 (1.50–4.92)	1.50	1.92	0.37 (1.73–2.10)
12	6	2	8.29	2.42 (7.08–9.50)	7.08	2.69	0.23 (2.57–2.80)	5	2	9.04	5.92 (6.08–12.00)	6.08	4.60	2.47 (3.37–5.83)
13	5	0						7	1	7.83	0.00 (7.83)	7.83	1.95	0.00 (1.95)
14	5	0						3	0					
16	7	0						10	1	8.00	0.00 (8.00)	8.00	3.23	0.00 (3.23)
17	10	0						10	2	7.50	0.00 (7.50)	7.50	2.60	0.00 (2.60)
19	7	0						5	2	6.32	1.97 (5.33–7.30)	5.33	3.07	2.73 (1.70–4.43)
21	8	0						8	4	8.04	7.07 (4.42–11.48)	4.42	3.12	5.53 (1.73–7.27)
22	5	1	7.67	0.00 (7.67)	7.67	2.30	0.00 (2.30)	8	1	5.13	0.00 (5.13)	5.13	1.70	0.00 (1.70)
23	5	0						6	3	7.65	3.50 (6.30–9.80)	7.65	2.43	0.97 (1.60–2.57)
TOTAL	7	0	7.67	0 (3.33–9.50)	7,08	2.57	2.20 (2.20–4.40)	7	2	7.00	13.25 (1.50–14.75)	6,54	2.68	14.83 (1.33–16.17)

GIP: gluten immunogenic peptides; LFIA: lateral flow immunoassay.

**Table 2:** Urine GIP detection in 3-hour periods after 50 mg and 2 g of gluten intakes.

	50 mg Gluten			2 g Gluten		
Time	Participants	GIP+ Participants	GIP [ng/mL]	Participants	GIP+ Participants	GIP [ng/mL]
h	*n*	*n*	Median (Range)	*n*	*n*	Median (Range)
0–3	15	0	0.00 (0)	15	1	0.00 (0–2.10)
3–6	15	1	0.00 (0–4.40)	15	10	1.70 (0–16.17)
6–9	16	3	0.00 (0–2.80)	18	14	2.57 (0–13.20)
9–12	18	1	0.00 (0–2.57)	16	8	0.00 (0–4.83)
12–15	18	0	0.00 (0)	17	7	0.00 (0–3.37)
15–18	8	0	0.00 (0)	6	0	0.00 (0)
18–21	6	0	0.00 (0)	4	0	0.00 (0)
21–24	14	0	0.00 (0)	12	0	0.00 (0)

GIP: gluten immunogenic peptides.

**Table 3:** Estimated diagnostic sensitivity of the methods in a specific range of time and with different sample collections.

LFIA Sensitivity in Urine			
	50 mg Gluten/2 g Gluten		
Time (h)	1 Sample (%)	2 Samples (%)	3 Samples (%)
3–6	6/47.8	11.6/72.8	16.9/85.8
6–9	13/72	24.3/92.2	34.1/97.8
9–12	4/57.1	7.8/81.6	11.5/92.1
3–12	7/59	13.5/83.2	19.6/93.1
